# Forensic evidence from a 30-year study of non-thrombotic pulmonary embolism

**DOI:** 10.1007/s00414-026-03938-3

**Published:** 2026-08-05

**Authors:** Stefano Tambuzzi, Guendalina Gentile, Michele Boracchi, Matteo Marchesi, Riccardo Zoia

**Affiliations:** 1 Laboratorio di Istopatologia Forense e Microbiologia Medico Legale - Sezione di Medicina Legale e delle Assicurazioni dell’Università degli studi di Milano, Milano, Italy; 2 S.S. Medicina Legale del Polo Ospedaliero ASST Papa Giovanni XIII, Piazza OMS 1, Bergamo, 24127 Italy

**Keywords:** Non-thrombotic pulmonary embolism, Bone marrow embolism, Fat embolism, Septic embolism, Tumor embolism, Amniotic embolism, Forensic pathology, Autopsy, Sudden death.

## Abstract

Non-thrombotic pulmonary embolism (NTPE) represents a heterogeneous and underrecognized group of conditions characterized by the obstruction of the pulmonary vasculature by non-thrombotic material, often associated with complex pathogenetic mechanisms and non-specific clinical presentations. Despite their potential to cause sudden and unexpected death, comprehensive forensic studies on NTPE remain limited. This study presents a 30-year retrospective forensic analysis (1993–2024) of autopsy cases performed at the Institute of Legal Medicine of Milan, aimed at identifying and characterizing deaths attributable to NTPE. Among 325 fatal pulmonary embolisms, 32 cases (9.8%) were classified as NTPE. Bone marrow embolism was the most frequent subtype (37.5%), followed by septic (25%), fat (21.8%), and amniotic fluid embolism (9.4%), with single cases of air and neoplastic embolism. NTPE occurred predominantly in traumatic and iatrogenic settings and were frequently associated with surgical or invasive procedures. Macroscopic findings were often non-specific, whereas histopathological examination proved decisive for diagnosis, frequently requiring targeted histochemical techniques to identify the nature of the embolic material. These findings highlight the intrinsic diagnostic complexity of NTPE and the risk of under recognition in the absence of systematic post-mortem investigation. In this context, a comprehensive forensic autopsy approach remains essential for the accurate identification and classification of these entities. Increased awareness of NTPE, together with the integration of histopathological and ancillary analyses, may contribute to improving diagnostic accuracy and to a more reliable interpretation of sudden and unexpected deaths.

## Introduction

Non-thrombotic pulmonary embolism (NTPE) [1] is considerably less common than pulmonary thrombotic embolism (PTE) and consists of the partial or complete occlusion of the pulmonary circulation by a wide range of materials [2], both organic and inorganic [3]. These include cellular elements (adipocytes, hematopoietic cells, trophoblastic or neoplastic cells), amniotic fluid, bacteria, fungi, foreign material, and gas [4, 5]. Due to the heterogeneity of the embolic agents involved, NTPE encompasses a broad spectrum of clinical presentations, reflecting a complex and multifactorial pathogenesis. Unlike thrombotic embolism, its effects are not solely mechanical but also involve severe pulmonary and systemic inflammatory responses, making the underlying mechanisms significantly more complex [6]. NTPE represents a major diagnostic challenge. In contrast to thrombotic embolism, the emboli are frequently not detectable on computed tomography pulmonary angiography (CTPA) [2], requiring radiologists to rely on indirect imaging findings [7–9]. Clinically, NTPE may present with highly variable and often non-specific manifestations, ranging from acute and dramatic conditions such as acute respiratory distress syndrome to more insidious or advanced presentations that may culminate in sudden death, with important medico-legal implications. However, large autopsy-based series specifically addressing NTPE remain scarce, and available evidence is mainly limited to isolated case reports or small clinical series. In this context, the present study provides one of the largest forensic analyses of NTPE, allowing a systematic characterization of its different forms within a medico-legal framework. A retrospectively examination of the autopsy case series of the Institute of Legal Medicine of Milan over a 30-year period (1993–2024) was performed, identifying all cases in which death was attributed to NTPE. For each category of NTPE, we provide a comprehensive overview supported by a targeted review of the literature and by illustrative cases with corresponding histopathological findings. The aim of this study is to highlight the forensic-pathological features of NTPE, the underlying lethal mechanisms associated with each condition, the related risk factors, and the key histopathological findings required for accurate forensic diagnosis and interpretation of these rare but clinically significant entities. In addition, a focused review of the literature was performed to include and discuss reported cases of NTPE that were not observed in our autopsy series, in order to provide a more comprehensive overview of the full spectrum of this condition.

## Materials and methods

From the institutional database of the Institute of Legal Medicine of Milan, we retrospectively selected all forensic autopsy cases recorded between January 1993 and December 2024 (*n* = 28,802), identifying those in which non-thrombotic pulmonary embolism (NTPE) had been established as the cause of death. The diagnosis of NTPE was based on combined macroscopic and histopathological findings, with identification of non-thrombotic embolic material within the pulmonary circulation. Cases of thrombotic pulmonary embolism and other causes of death were excluded.

For each case, all available data were collected, including demographic characteristics (sex and age), circumstantial information, clinical history (including comorbidities and pharmacological treatments), and the state of body preservation at the time of autopsy. Histological analyses were performed according to standard forensic practice, when authorized by the Judicial Authority. Lung sampling was performed according to a standardized topographic protocol, including at least five tissue blocks, one from each pulmonary lobe. Samples were collected in a standardized sequence, beginning with the left upper lobe, followed by the left lower lobe, and then the right upper, middle, and lower lobes. The tissue blocks were obtained in progressively decreasing size, thereby allowing immediate identification of the anatomical lobe of origin during histopathological examination. In addition, both pulmonary hila were routinely sampled, with the left hilar specimen being larger than the right hilar specimen. Whenever areas of pulmonary infarction were identified macroscopically, additional tissue blocks were taken from the transition zone between the infarcted (hemorrhagic-necrotic) area and the adjacent viable lung parenchyma, and the sampling site was carefully documented.

To ensure a comprehensive evaluation of the different forms of NTPE, a structured literature review was also conducted using major electronic databases (PubMed, Scopus, MEDLINE, and Web of Science). The search strategy was based on combinations of the following free-text terms, using the Boolean operator “AND”: “non-thrombotic pulmonary embolism,” “unexpected death,” “forensic medicine,” “autopsy,” “bone marrow embolism,” “air embolism,” “septic pulmonary embolism,” “tumor embolism,” “fat embolism,” and “amniotic embolism.”

Preference was given to recent publications, while earlier studies were included when considered relevant or frequently cited. The reference lists of selected articles were also screened to identify additional pertinent studies. This review was not conducted as a systematic review but aimed to provide a comprehensive contextual framework for the interpretation of the observed cases.

Finally, for each NTPE category, representative cases were selected and are presented as illustrative examples supported by histopathological findings.

### Ethical considerations

The study did not involve living human participants or animals. All subjects included in this study underwent court-ordered medico-legal autopsy at the Institute of Legal Medicine of Milan to determine the cause of death. The collection of biological samples and all subsequent forensic analyses were authorized by the Public Prosecutor within the framework of a judicial investigation, in accordance with the Italian Mortuary Police Regulation (D.P.R. 10 September 1990, No. 285). The study was conducted in accordance with national legislation, the guidelines of the Italian National Bioethics Committee, and the principles of the Declaration of Helsinki. All data were retrospectively collected, fully anonymized, and processed in compliance with the Italian Data Protection Authority provisions (Official Gazette No. 72, 26 March 2012) and the General Data Protection Regulation (EU) 2016/679. According to the regulations governing judicial autopsies in Italy, retrospective studies based on data obtained from court-ordered autopsies do not require approval by an Institutional Ethics Committee; therefore, ethical approval was waived.

## Results

A total of 325 cases of pulmonary embolism were identified from the institutional database. Of these, 293 cases were attributed to fatal pulmonary thrombotic embolism, whereas 32 cases (9.8%) were classified as non-thrombotic pulmonary embolism (NTPE).

**Fig. 1:**
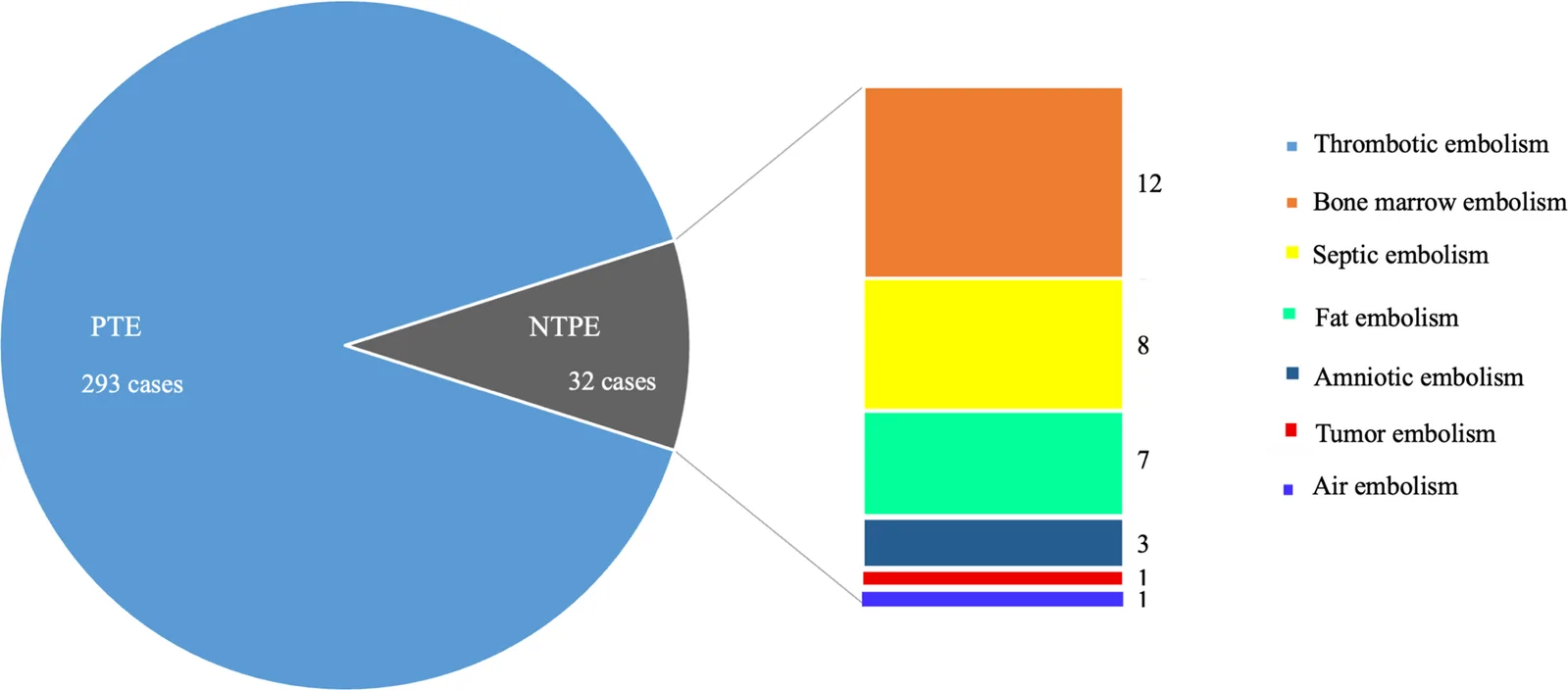
Graphical representation of fatal pulmonary embolism cases, showing the distribution between thrombotic pulmonary embolism (PTE) and non-thrombotic pulmonary embolism (NTPE) over the study period. The different NTPE subtypes are presented in decreasing order of frequency

Among NTPE cases, bone marrow embolism (with or without an associated fat component) was the most frequent finding (*n* = 12), followed by septic embolism (*n* = 8), fat embolism (*n* = 7), amniotic fluid embolism (*n* = 3), and isolated cases of air embolism (*n* = 1) and neoplastic embolism (*n* = 1) (Fig. 1).

All bodies were in a good state of preservation, with a post-mortem interval (PMI) not exceeding 7 days between discovery and autopsy.

Overall, trauma-related and iatrogenic conditions accounted for the majority of NTPE cases, highlighting the predominant role of external or procedure-related triggers in the pathogenesis of these entities.

### Bone marrow embolism (BME)

Bone marrow embolism (BME) is a rare condition traditionally associated with bone trauma [10], particularly involving bones rich in red marrow [11].

It is characterized by the entry into the systemic circulation - predominantly the small pulmonary arteries [12] - of bone marrow elements, including mature and immature erythrocytes, lymphoid and myeloid cells, adipocytes, and megakaryocytes. BME most commonly develops in traumatic or mechanically induced conditions. The principal triggering events include fractures [12], contusions even in the absence of fractures [12], and situations associated with increased intramedullary pressure, such as displacement of unstable bone fragments or surgical manipulation of the medullary cavity during orthopedic or intraosseous procedures. Additional trauma-related or iatrogenic causes include cardiopulmonary resuscitation [12–14] and electrocution [15]. The likelihood of BME is influenced by the severity and type of injury, with higher risk observed in multiple or unstable fractures and in closed fractures where intramedullary pressure may rise more significantly. Alongside these, a number of non-traumatic conditions have also been associated with BME. These include hematological diseases [16–18], seizures [19], and, more rarely, cases occurring in the absence of any identifiable traumatic trigger [20]. Furthermore, BME has been described as a distinct entity in unusual locations, such as the coronary circulation [21].

Clinically, BME typically develops within 12–72 h after trauma [22], although variability may occur depending on the underlying condition. When bone marrow elements enter the bloodstream through damaged vascular structures at the site of injury, they may embolize to the lungs, systemic circulation, and distant organs [23], leading to obstruction of pulmonary vessels and impairment of respiratory function [24]. Clinical manifestations are often non-specific and may include respiratory distress, neurological symptoms, and petechial rash, contributing to frequent under recognition [1].

From an imaging perspective, radiological findings are generally non-specific. Chest radiography may demonstrate diffuse bilateral pulmonary infiltrates, whereas computed tomography (CT) typically reveals bilateral ground-glass opacities or patchy areas of consolidation. Because bone marrow emboli predominantly involve the pulmonary microcirculation, imaging is often non-diagnostic, and definitive diagnosis frequently relies on histopathological examination [2].

BME frequently coexists with fat embolism (FE), a related but distinct entity that may arise not only from bone injury but also from extraosseous sources such as liposuction [19]. The two conditions share overlapping pathogenetic mechanisms and both histopathological and radiological features [25], which may complicate differential diagnosis. If unrecognized, this process may progress to fat embolism syndrome (FES), in which lipase-mediated release of free fatty acids induces endothelial damage, edema, and vasogenic hemorrhage [19, 26] ultimately leading to severe pulmonary dysfunction. Definitive diagnosis is most often established post-mortem through histopathological examination [19], as clinical suspicion is frequently low. In autopsy series, BME has been reported in approximately 16% of cases [27]. From a medico-legal perspective, bone marrow embolism represents a reliable marker of vitality, as the embolization of bone marrow elements requires active circulation at the time of injury. The identification of hematopoietic precursor cells, particularly megakaryocytes, and occasionally microscopic bone fragments within the pulmonary vasculature confirms ante-mortem vascular dissemination following skeletal trauma. This finding also contributes to reconstructing the sequence of traumatic events [2].

#### Results of the retrospective evaluation

Bone marrow embolism was documented in 12 cases (37.5% of all NTPE), representing the most frequent subtype. The cohort included 6 females aged between 50 and 86 years (mean 69.1 years) and 6 males aged between 39 and 62 years (mean 51.5 years).

Trauma-related conditions predominated, including polytrauma from traffic accidents (*n* = 4), involving multiple body regions and skeletal injuries, and accidental falls (*n* = 2) resulting in fractures of the upper and/or lower limbs (predominantly long bones). Four additional cases occurred in patients with prolonged immobilization (bedridden or impaired mobility) complicated by known or occult pathological fractures (*n* = 4). One case involved a patient with cerebral lymphoma and bone involvement who was found deceased at home, and one followed intentional assault.

The interval between the initial clinical event (trauma, hospitalization, or onset of prolonged immobilization) and death ranged from 7 h to 120 days. The longest interval occurred in a bedridden patient with prolonged immobilization and an underlying skeletal lesion, without evidence of any more recent traumatic or surgical event. Comorbidities were frequent and included cardiovascular diseases, obesity, diabetes, neoplasms, senile dementia, and osteoporosis. Reported pharmacological treatments included anti-inflammatory drugs, statins, diuretics, antigout agents, antihypertensives, anticoagulants, anti-osteoporotic drugs, and insulin.

Histopathological examination allowed attribution of death to isolated bone marrow embolism in 9 cases and to bone marrow embolism associated with a fat component in 3 cases.

#### Illustrative cases

##### Case no. 1

A 50-year-old woman (90 kg, 171 cm) accidentally slipped at home, sustaining a fracture of the right anterior fibular malleolus with indication for surgical treatment. Six days after the trauma, she underwent open reduction and internal fixation with plate and screws. Nine days after surgery, she died suddenly.

**Fig. 2:**
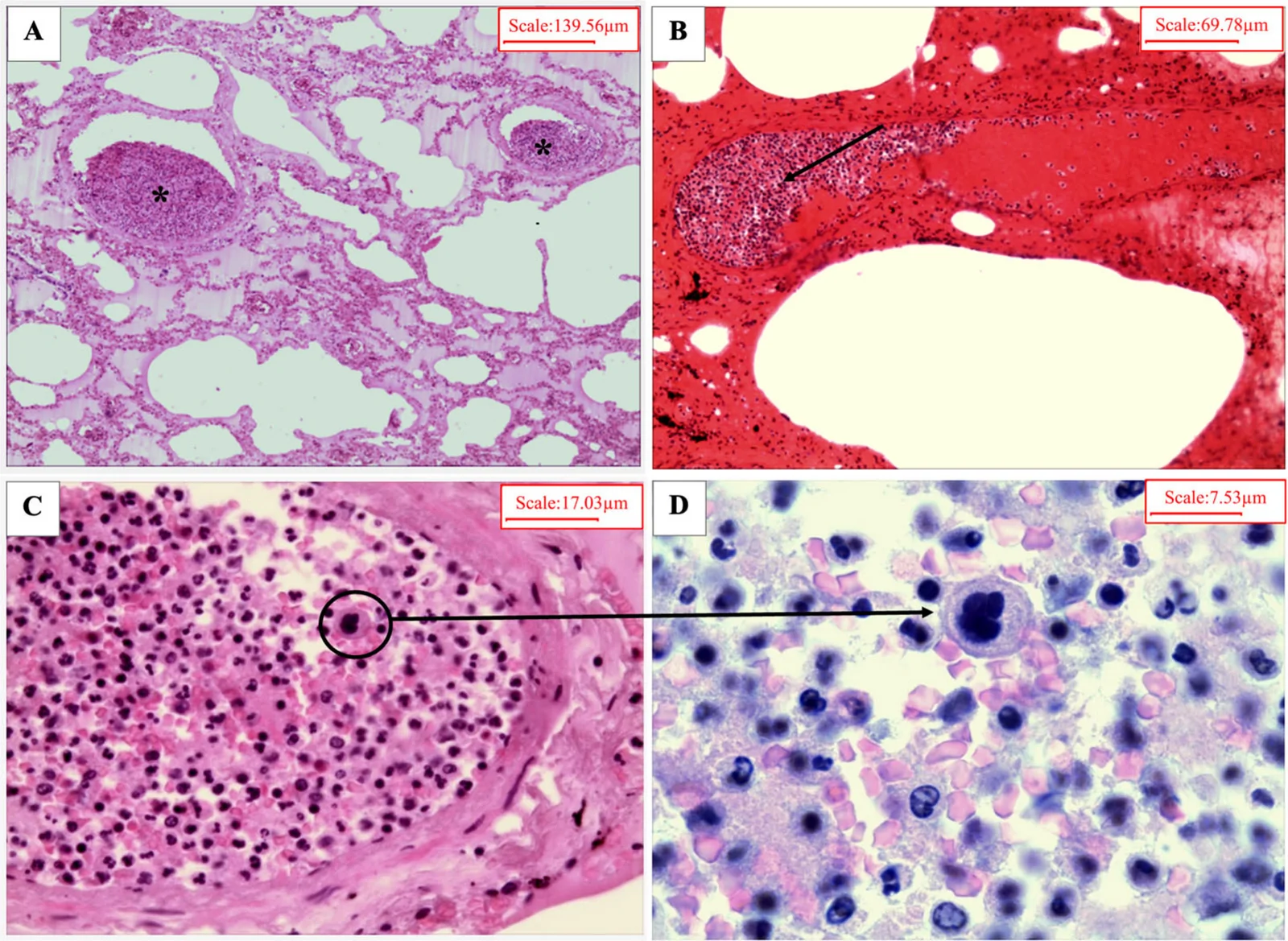
Microscopic images of lung sections showing isolated bone marrow embolism characterized by hematopoietic elements. In A (H&E, 50×): multiple foci of bone marrow emboli (asterisks). In B (H&E, 100×): area of pulmonary infarction with a bone marrow embolus (arrow). In C (H&E, 400×): higher magnification of a bone marrow embolus showing a megakaryocyte and hematopoietic precursors. In D (May–Grünwald–Giemsa, 1000×): detailed visualization of the megakaryocyte

At autopsy, the lungs appeared enlarged and increased in weight (right lung 530 g; left lung 420 g), markedly congested, with diffuse subpleural petechiae. Microscopic examination revealed numerous bone marrow emboli occluding small-caliber pulmonary arterial vessels, extending into the parenchyma up to the subpleural regions (Fig. 2A). An acute hemorrhagic infiltrate with rupture of alveolar septa, consistent with pulmonary infarction in a hemorrhagic phase, was identified. At the periphery and within the infarcted areas, multifocal bone marrow emboli with vascular lumen occlusion was observed (Fig. 2B). In larger vessels, embolic material appeared partially adherent to the vascular wall. The emboli showed well-preserved bone marrow cellularity, with recognizable elements of both myeloid and erythroid lineages, including megakaryocytes (Fig. 2C and D). Additional pulmonary findings included focal intra-alveolar microhemorrhages and chronic remodeling of small-caliber pulmonary arteries.

At the conclusion of all investigations, the cause of death was identified as massive pulmonary embolism due to bone marrow emboli.

##### Case no. 2

A 39-year-old man (86 kg, 171 cm) sustained cranioencephalic trauma and a femoral fracture following a physical assault and died in hospital 2 days later.

**Fig. 3:**
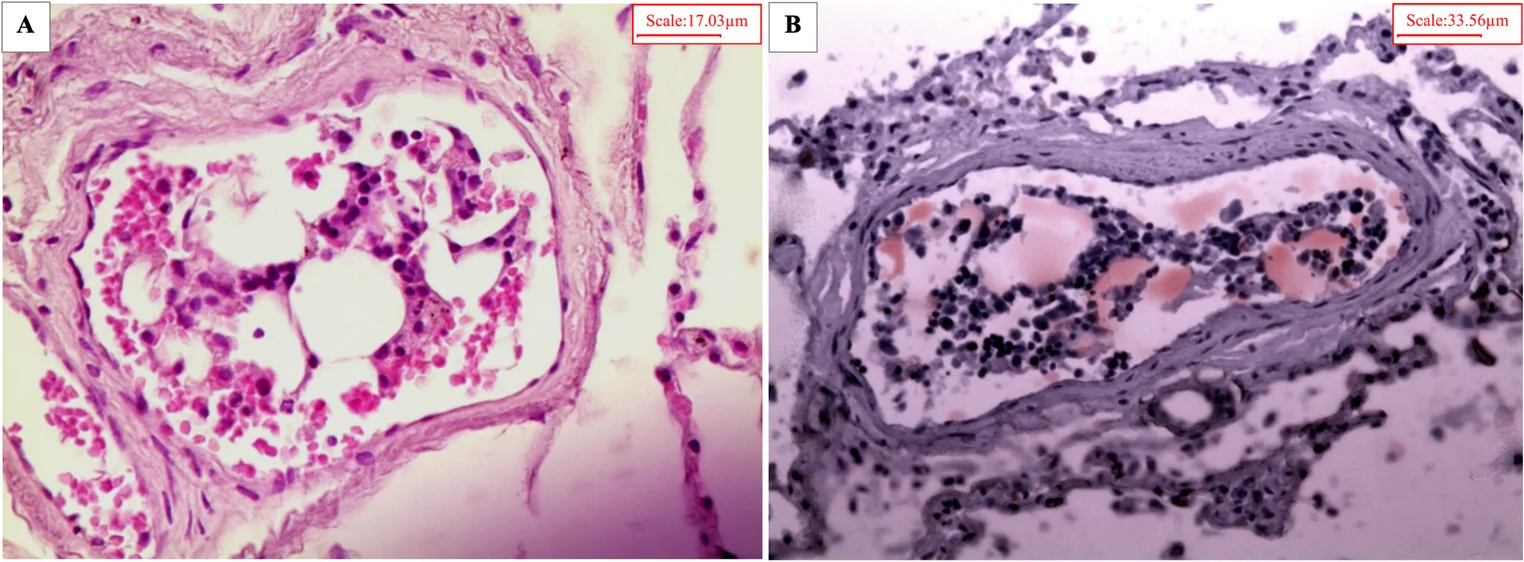
Microscopic images of lung sections showing bone marrow embolism associated with a concomitant fat component. Hematopoietic elements are demonstrated in A (H&E, 400×), whereas the associated fat emboli are highlighted by Oil Red O staining in B (200×)

At autopsy, the lungs appeared enlarged and increased in weight (right lung 590 g; left lung 490 g), congested, with focal subpleural petechiae. Microscopic examination revealed bone marrow emboli within small-caliber pulmonary vessels, occasionally extending to alveolar septal capillaries and subpleural areas, associated with a limited fat component (Fig. 3A and B). The associated fat component was confirmed by Oil Red O staining performed on samples from all pulmonary lobes. Additional pulmonary findings included pulmonary edema. At the conclusion of all investigations, the cause of death was identified as pulmonary embolism due to bone marrow emboli with an associated fat component.

### Septic pulmonary embolism (SPE)

Septic pulmonary embolism (SPE) arises as a complication of a primary extrapulmonary infectious focus - such as cardiac valves [28], tonsils and dental infections [29], pelvic sites [30], or intravascular devices including long-term central venous catheters and pacemaker leads [31, 32] - in which infected thrombi containing microorganisms embedded in fibrin are formed. These septic thrombi may detach and migrate through the venous circulation to the pulmonary arteries, where they lodge in distal branches [33], leading to vascular occlusion and secondary complications such as pulmonary infarction, metastatic abscesses, empyema, bronchopleural fistulas, sepsis, and death [34]. Although historically considered rare, SPE is now increasingly recognized as an underestimated and clinically challenging condition [35, 36], with a rising incidence [33], particularly among intravenous drug users [37] and immunocompromised individuals, including pediatric patients [1]. A distinctive subtype is Lemierre’s syndrome, a rare complication of oropharyngeal infection [38], characterized by septic thrombophlebitis of the internal jugular vein with pulmonary involvement reported in up to 97% of cases, frequently associated with abscess formation and empyema [39]. Clinically, SPE presents with a broad and often non-specific spectrum, ranging from pneumonia-like symptoms (fever, dyspnea, cough, hemoptysis, and pleuritic chest pain) to sepsis and multiple organ dysfunction syndrome [8, 40]. Diagnosis remains challenging [41], as imaging findings may overlap with those of thrombotic pulmonary embolism. Computed tomography (CT) is the preferred diagnostic modality [41], typically showing multiple bilateral nodules, ground-glass opacities, or wedge-shaped cavitary lesions [42], which may vary according to the causative pathogen [43]. From a microbiological perspective, SPE may be caused by bacterial or fungal pathogens.

Fungal embolism is typically associated with opportunistic organisms such as *Mucor*, *Aspergillus*, and *Candida*, which exhibit angioinvasive and tissue-destructive properties [44]. These infections may remain localized [45–48] or evolve into disseminated forms via hematogenous spread [44, 49], leading to septic thromboembolism, pulmonary infarctions, and visceral abscesses [50], often with an acute or fulminant clinical course [51]. Such cases are predominantly observed in immunocompromised individuals, particularly those with hematological malignancies [1, 52], and may be favored by conditions such as diabetes mellitus with ketoacidosis, burns, glomerulonephritis, gastroenteritis, and organ transplantation [45, 53].

Bacterial embolism more commonly involves Gram-positive organisms, particularly methicillin-sensitive and methicillin-resistant *Staphylococcus aureus*, as well as *Streptococcus viridans* [40, 54]. Among Gram-negative bacteria, *Fusobacterium*, *Pseudomonas aeruginosa*, ESBL-producing Enterobacteriaceae, carbapenem-resistant *Acinetobacter baumannii*, vancomycin-resistant *Enterococcus*, and *Klebsiella pneumoniae* have been reported, the latter frequently associated with pyogenic liver abscesses complicated by SPE [55, 56].

Additional rare cases include embolism caused by *Proteus mirabilis*, a biofilm-forming organism associated with foreign materials such as urinary and central venous catheters [57, 58], which may lead to systemic infection but only rarely to septic pulmonary embolism [59]. From a medico-legal perspective, septic pulmonary embolism represents a reliable marker of vitality, as both embolization and the associated inflammatory response require active circulation. The presence of septic vasculitis, characterized by neutrophilic infiltration of the vascular wall with bacterial invasion, confirms ante-mortem hematogenous dissemination of infection and contributes to reconstructing the temporal evolution of the infectious process and the mechanism of death [2].

#### Results of the retrospective evaluation

Septic pulmonary embolism was documented in 8 cases (25% of all NTPE), including 5 fungal (62.5%) and 3 bacterial cases (37.5%).

Fungal embolism involved 2 females (aged 73 and 74 years) and 3 males (aged 59–79 years; mean 68 years). All patients died in hospital: four after a hospitalization period ranging from 6 to 43 days, and one during a bronchoscopic procedure performed for the evaluation of a pulmonary nodular lesion. The clinical contexts included recent cardiac surgery (mitral valve revision/replacement), abdominal pathology complicated by bowel perforation and enterorrhagia, severe metabolic acidosis with renal failure, and recent orthopedic surgery with displacement of osteosynthesis. Underlying conditions were frequently present and included neoplasms (*n* = 3), diabetes, valvular disease, and cardiovascular disorders. Reported treatments included chemotherapeutic agents, immunosuppressants, insulin, and antihypertensive drugs. In all cases, post-mortem histological examination identified fungal embolism as the cause of death, due to *Aspergillus* (*n* = 4) or *Candida* (*n* = 1).

Bacterial embolism involved 1 female (55 years) and 2 males (67 and 73 years). The female was found dead at home, whereas the two male patients died in hospital 63 and 24 days after admission, respectively. The 67-year-old male had a history of arterial hypertension and lung neoplasm and had sustained polytrauma in a traffic accident, subsequently complicated by pressure sores and pneumonia. The 73-year-old male, affected by HCV infection, hypertension, and pancreatic head carcinoma, had undergone pancreaticoduodenectomy, complicated by biliary fistula and sepsis. In all cases, post-mortem histological examination allowed attribution of death to septic embolism caused by *Staphylococcus* spp. (*n* = 2) and *Escherichia coli* (*n* = 1).

#### Illustrative cases

##### Caso no. 1

A 74-year-old woman (90 kg, 154 cm), with a history of aortic valve prosthesis implantation performed 17 years earlier, was admitted for revision/replacement due to recent cardiac complications. Following cardiac surgery, initially uneventful, she developed fever and was found dead in her hospital bed 13 days later.

**Fig. 4:**
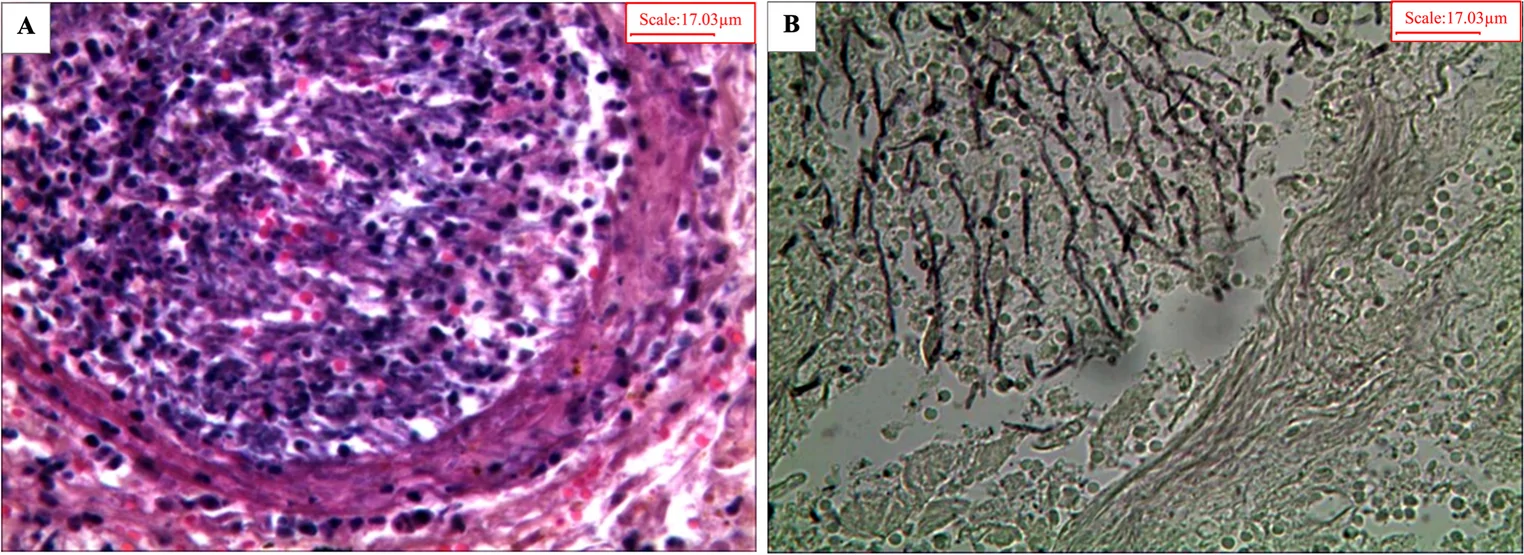
Microscopic images of lung sections showing fungal septic embolism. In A (H&E, 400×): fungal thrombosis in small- to medium-sized vessels, with thrombi containing neutrophils incorporating fungal elements. In B (Grocott’s methenamine silver, 400×): fungal elements highlighted by silver staining, protruding into the vascular lumen and associated with neutrophils. The background was not counterstained with Light Green to enhance visualization of fungal structures

At autopsy, the lungs appeared enlarged and increased in weight (right lung 480 g; left lung 390 g), markedly congested and edematous, with scattered subpleural petechiae. Microscopic examination revealed widespread fungal septic emboli occluding small-caliber pulmonary vessels, associated with multiple microabscesses composed of neutrophilic granulocytes containing fungal elements (Fig. 4A), in the context of marked pulmonary edema. Grocott’s methenamine silver staining confirmed the presence of fungal elements with yeast-like morphology, highlighted in black (Fig. 4B). Numerous recent intra-alveolar and subpleural hemorrhages were also observed. At the conclusion of all investigations, the cause of death was identified as fungal pulmonary embolism due to *Candida*. The attribution of death was based on the extensive fungal occlusion of the pulmonary microvasculature associated with multiple septic pulmonary microabscesses. Neither the autopsy nor the histopathological examination revealed evidence of disseminated fungal infection or other alternative acute causes of death, supporting pulmonary septic embolism as the predominant fatal mechanism.

##### Case no. 2

A 55-year-old woman (57 kg, 167 cm), with a history of long-standing drug abuse (> 30 years), HIV infection, alcoholism, and smoking, was found dead at home by law enforcement officers alerted by neighbors.

**Fig. 5:**
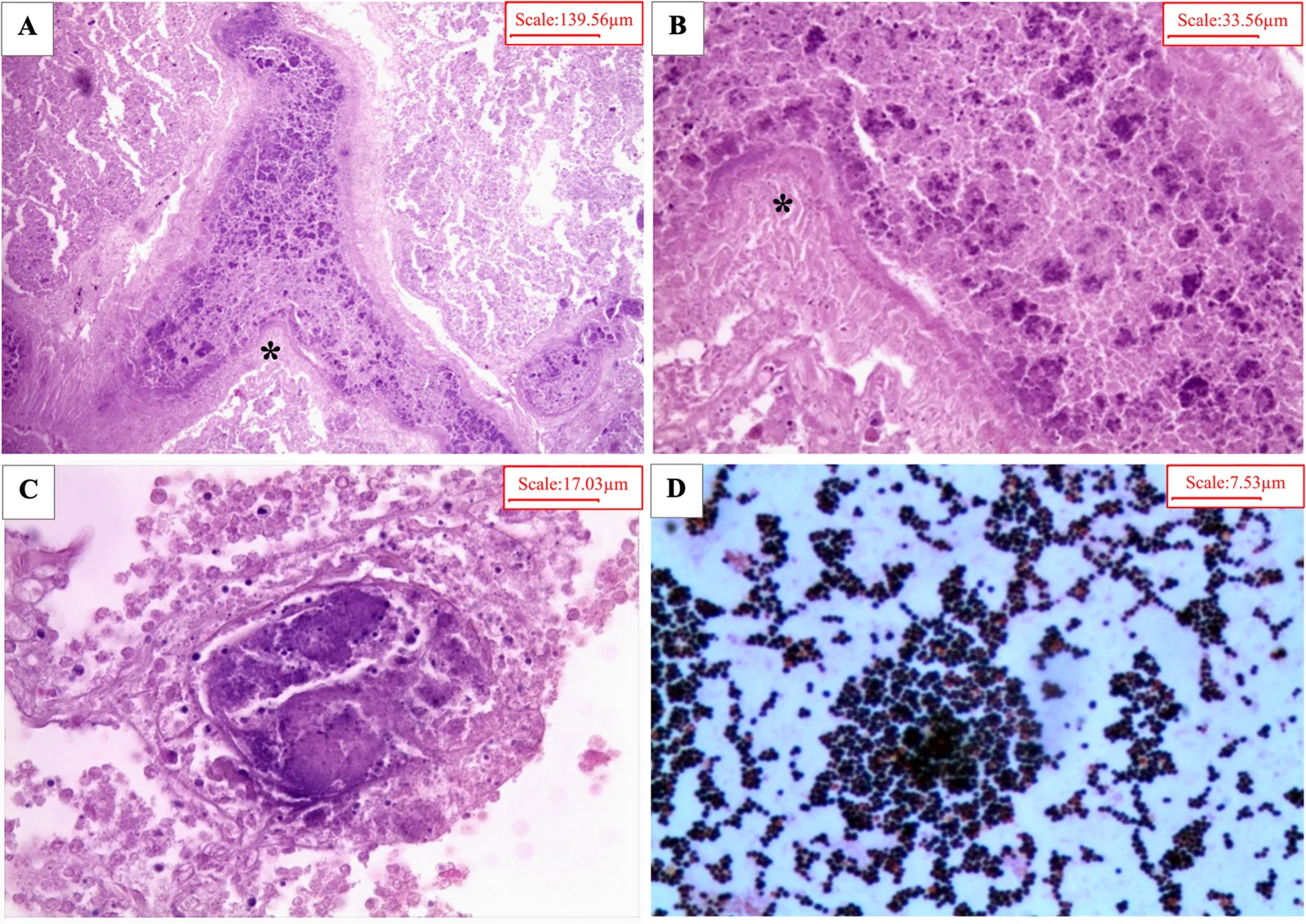
Microscopic images of lung sections showing bacterial septic embolism. In A (H&E, 50×): multiple bacterial septic emboli occluding small- to medium-sized pulmonary vessels. In B-C (H&E, 200× and 400×): higher magnification of bacterial septic emboli. In D (Gram stain, 1000×): Gram-positive coccoid bacterial aggregates arranged in clusters within the lumen of a pulmonary vessel (septic embolus), consistent with Staphylococcus spp

At autopsy, the lungs appeared enlarged and increased in weight (right lung 430 g; left lung 385 g), markedly congested and edematous, with scattered subpleural petechiae. The pulmonary parenchyma showed multiple cavitary lesions containing purulent material. Microscopic examination revealed massive bacterial embolization of pulmonary vessels, associated with multifocal abscess formation involving the lungs (Fig. 5A and C), as well as direct extension by contiguity to the adventitia of the aorta and the outer third of the myocardial wall, in association with fibrinous pericarditis. Gram staining demonstrated Gram-positive bacterial aggregates consistent with *Staphylococcus* spp. (Fig. 5D).

At the conclusion of all investigations, the cause of death was identified as septic pulmonary embolism due to *Staphylococcus* spp. The attribution of death was based on the massive bacterial embolization of the pulmonary vasculature associated with multifocal pulmonary abscesses. Although the inflammatory process extended by contiguity to the adventitia of the aorta and the outer third of the myocardial wall, with associated fibrinous pericarditis, neither the autopsy nor the histopathological examination demonstrated septic emboli in distant organs or other alternative acute causes of death, supporting septic pulmonary embolism as the predominant fatal mechanism.

### Fat embolism (FE)

Fat embolism (FE) is characterized by the presence of fat globules within the circulation, which, after entering the venous and/or arterial system and reaching the pulmonary vasculature, cause vascular obstruction. Fat emboli may also involve capillary beds in other organs, including the brain, myocardium, and kidneys [60], potentially leading to fat embolism syndrome (FES) [61], a severe clinical condition defined by the triad of respiratory distress, neurological impairment, and petechial rash [23].

Although the pathophysiology of FE is not fully understood, two main forms are recognized:

1. *Endogenous (non-traumatic) form*, also referred to as microscopic fat embolism [41], resulting from the obstruction of small vessels by circulating fat droplets. This form is typically associated with underlying pathological conditions such as sickle cell anemia [62], pancreatitis [2], hepatitis and hepatocellular carcinoma [63], fulminant osteomyelitis, diabetes mellitus, and nephrotic syndrome. Several mechanisms have been proposed to explain its development, including: (i) disturbances in lipid metabolism leading to increased levels of free fatty acids with toxic effects on pulmonary endothelium; (ii) inflammatory cascade activation involving C-reactive protein; (iii) aggregation of chylomicrons [64]; (iv) involvement of very-low-density lipoproteins [65].
2. *Exogenous (traumatic) form*, also known as macroscopic fat embolism [41], characterized by mechanical obstruction of pulmonary vessels by larger fat globules. This form is most commonly associated with traumatic events, particularly fractures of bones rich in adipose tissue, such as long bones and the pelvic girdle. Given the presence of bone marrow in these structures, a concomitant medullary embolic component may coexist, as discussed in the section on bone marrow embolism.

FE may also occur in the absence of fractures, following extensive soft tissue contusions or burns, and may represent a frequent but often underestimated finding at autopsy [66]. Additional cases have been described as complications of cosmetic procedures such as liposuction [67] and lipoinjection [68], as well as various medical interventions, including intra-arterial chemotherapy with cisplatin, blood transfusion, organ transplantation (bone marrow or kidney), parenteral lipid infusion, coronary bypass surgery, high-dose corticosteroid therapy, inhalational anesthesia, radiological procedures using oily contrast agents, and orthopedic surgery [69]. Advanced resuscitation maneuvers may also lead to FE [61], particularly in pediatric patients undergoing intraosseous access [70], likely due to increased intramedullary pressure and microfractures facilitating the entry of fat into the circulation.

Clinically, FE should be suspected in patients presenting, typically within 24–72 h after injury, with respiratory symptoms (acute respiratory distress), neurological manifestations (altered consciousness, lethargy, coma), and cutaneous signs (petechiae) [71]. From an imaging perspective, chest radiography is generally non-specific and may reveal diffuse bilateral pulmonary infiltrates resembling acute respiratory distress syndrome (ARDS) [2]. Computed tomography (CT) typically demonstrates bilateral ground-glass opacities, areas of consolidation, poorly defined centrilobular nodules, and, occasionally, fat-attenuation filling defects within the pulmonary arteries [41]. However, because fat emboli predominantly involve the pulmonary microcirculation, CT pulmonary angiography may be negative despite severe clinical manifestations.

In forensic practice, FE is of particular relevance but remains markedly underdiagnosed [72], as macroscopic findings are often non-specific or absent [73]. Histological examination is therefore essential for diagnosis. From a medico-legal perspective, fat embolism represents a reliable marker of vitality, as the presence of fat emboli within the pulmonary circulation confirms ante-mortem embolization requiring active circulation [74]. Microscopic detection requires lipid-specific staining techniques, such as Sudan stains or Oil Red O. Quantification of fat embolism in pulmonary tissue can be performed using standardized scoring systems, such as the Falzi’s classification [75], which scores embolic burden into four levels (Table 1). Although score 3 is generally regarded as the histopathological pattern most consistently associated with fulminant fatal fat embolism syndrome, score 2 embolization already reflects diffuse pulmonary microvascular involvement and may be associated with severe respiratory impairment. Therefore, the histopathological score should always be interpreted in conjunction with the clinical presentation, circumstantial evidence, and autopsy findings [76].

**Table 1:** Falzi’s scoring system for quantifying fat embolism in histological sections

Score	Number of Emboli Observed
0	Non-fat embolism, punctiform when present, sporadic, and not observed in all microscopic fields.
1	Mild fat embolism, sporadically present in all microscopic fields, with droplet-shaped fat globules.
2	Fat embolism with multiple globules, disseminated in every microscopic field, with “sausage-shaped” or rounded morphology.
3	“Massive” fat embolism, with horn-shaped globules, widely distributed and present in large numbers in every microscopic field.

#### Results of the retrospective evaluation

Fat embolism (FE) was documented in 7 cases (21.8% of all NTPE), almost exclusively affecting females aged 77–91 years, with a single younger case (26 years; mean age 75.3 years). Death occurred between 10 h and 8 days after the triggering event. In most cases (*n* = 4), FE followed polytrauma with fractures involving the vertebrae, pelvis, and long bones, sustained in traffic accidents. Additional cases included one death 10 h after hip arthroplasty, one following a femoral fracture treated surgically with death 22 h postoperatively, and one due to assault-related polytrauma with lower limb fractures (death after 12 h). Comorbidities were common and included cardiovascular disease, rheumatoid arthritis, gastric ulcer, asthma, osteoporosis, and hypertension. Reported treatments included anti-inflammatory drugs, anti-asthmatic agents, antihypertensives, antiplatelet agents, anticoagulants, and anti-osteoporotic therapy. In all cases, the cause of death was attributed to fat embolism based on histopathological examination, confirmed by specific histochemical staining techniques.

#### Illustrative case

An 82-year-old woman (91 kg, 156 cm), affected by severe coxofemoral osteoarthritis, underwent right hip arthroplasty. In the immediate postoperative period, her clinical condition progressively deteriorated, leading to death 10 h after surgery.

**Fig. 6:**
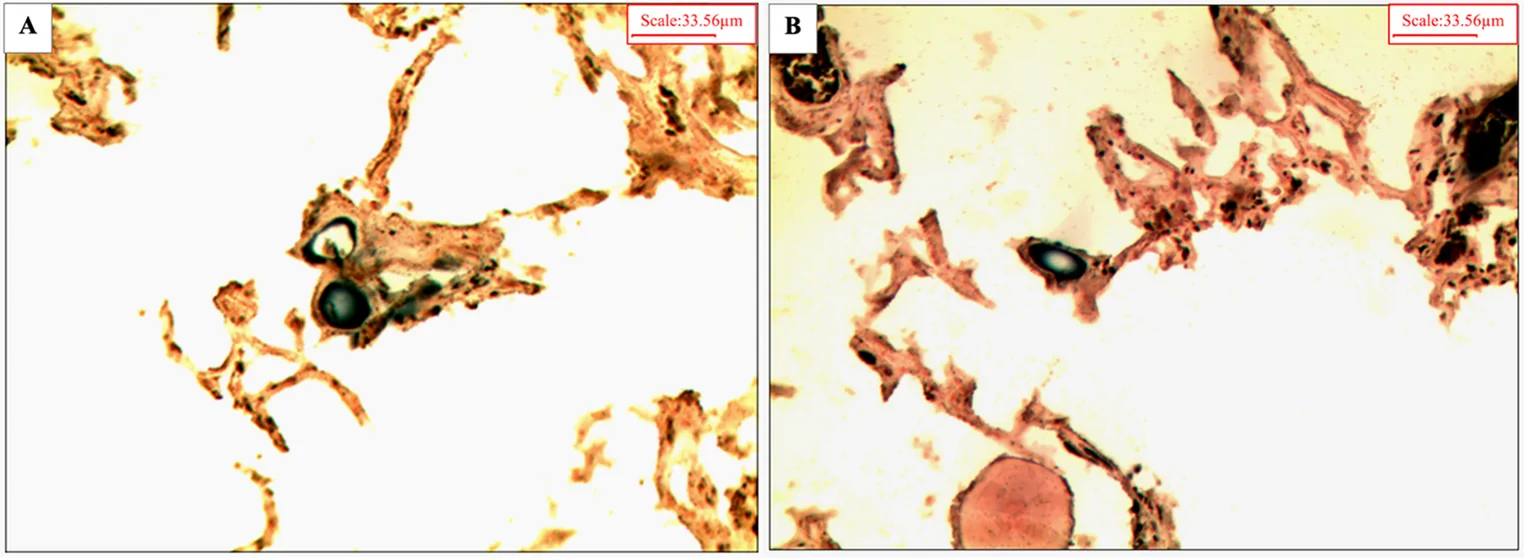
Microscopic images of lung sections showing fat embolism. Rounded embolic droplets are positive with Sudan Black B staining and appear black (A–B, 200×)

At autopsy, the lungs appeared enlarged and increased in weight (right lung 550 g; left lung 460 g), markedly congested, with numerous diffuse subpleural petechiae. Histopathological examination revealed diffuse pulmonary fat embolism, with dilated capillaries of the alveolar septa appearing regularly rounded and optically empty, demonstrated by Sudan Black B lipid staining (Fig. 6A and B), corresponding to score 2 according to the Falzi’s scoring system. Additional pulmonary findings included marked pulmonary edema.

At the conclusion of all investigations, the cause of death was identified as fat embolism. In the present case, the widespread score 2 pulmonary fat embolization, interpreted in conjunction with the close temporal relationship to hip arthroplasty, the rapid postoperative clinical deterioration, the severe pulmonary edema observed at autopsy, and the absence of alternative acute causes of death, supported the attribution of death to fat embolism.

### Amniotic fluid embolism (AFE)

Amniotic fluid embolism (AFE) is a rare but catastrophic obstetric condition characterized by the sudden entry of amniotic fluid into the maternal circulation [77, 78]. It typically occurs during pregnancy, labor (52.6–70%), delivery, or the immediate postpartum period [79, 80]. Several predisposing factors have been identified, including advanced maternal age (> 35 years), previous cesarean section, multiple pregnancy [81], instrumental delivery, labor induction, polyhydramnios, placental abruption, placenta previa, uterine rupture, and abdominal trauma [82, 83]. These conditions are often associated with disruption of the maternal–fetal interface [84], particularly at the level of the endocervix, lower uterine segment, endocervical veins, and placental insertion sites [85], facilitating the passage of amniotic fluid into the maternal bloodstream. Amniotic fluid contains fetal urine, desquamated epithelial cells, lanugo, vernix caseosa, mucin, and bile pigments derived from meconium [77]. Its entry into the maternal circulation may trigger a sudden and complex syndrome with high maternal [85–88] and fetal mortality [88], for which no effective preventive strategies are currently available [89, 90]. Despite a low incidence (approximately 1 in 40,000 deliveries) [91], maternal and perinatal mortality rates remain high, ranging from 20% to 60% [91], even under optimal clinical management [92]. Early recognition is therefore crucial for improving outcomes [93–96]. Clinically, AFE presents with sudden onset of respiratory distress, severe hypoxemia, pulmonary hypertension, and cardiovascular collapse, often accompanied by tachycardia and hypotension. Disseminated intravascular coagulation (DIC), occurring in up to 85% of cases, may develop with or without overt bleeding, along with seizures, multiple organ dysfunction syndrome, and cardiac arrest [97–99].

The pathogenesis of AFE is complex and not fully understood. The simple presence of fetal components in the maternal circulation is not sufficient to explain the syndrome, as such elements may also be detected in asymptomatic pregnant women [100, 101]. Current evidence suggests that, once fetal material crosses the maternal–fetal barrier, it triggers a predominantly immune-mediated response [85, 102], involving cytokine activation [103], pulmonary vasoconstriction, and diffuse microthrombosis induced by activation of the coagulation cascade [78]. This process is further amplified by an anaphylactoid reaction to fetal and placental antigens, supported by increased serum tryptase levels and pulmonary mast cell activation [104]. These mechanisms may explain the rapid and often fatal course of AFE, even in the absence of significant mechanical obstruction of the pulmonary vasculature [105]. Despite advances in current understanding, AFE remains a diagnosis of exclusion lacking universally accepted criteria [2, 85], and is typically defined by the abrupt onset of hypoxia and hypotension followed by coagulopathy, in temporal relation to labor or delivery, after exclusion of other potential causes [77]. From an imaging perspective, the radiological findings of amniotic fluid embolism (AFE) are non-specific and primarily serve to exclude alternative diagnoses, as the diagnosis remains essentially clinical. Chest radiography may demonstrate diffuse bilateral air-space opacities, whereas computed tomography (CT) typically reveals bilateral ground-glass opacities, interlobular septal thickening, and areas of consolidation, predominantly involving the lower lobes. However, these findings are not pathognomonic and should always be interpreted in conjunction with the clinical presentation [2]. Therefore, in fatal cases, imaging findings should be integrated with the clinical history and confirmed by post-mortem histopathological examination. In this context, autopsy plays a fundamental role in confirming the diagnosis and excluding alternative causes of death, and documenting intravital embolization through the integration of histopathological findings with the clinical and obstetric history, representing a key step in the forensic evaluation of suspected cases [77, 106].

#### Retrospective evaluation results

Amniotic fluid embolism (AFE) was documented in 3 cases (9.4% of all NTPE), aged 35–38 years (mean age 36.3 years). All patients died in hospital within 1–2 days after admission. Clinical presentation varied: one patient collapsed immediately after rupture of membranes with sudden loss of consciousness; one died during labor, presenting with abdominal pain and cough; and one died shortly after delivery, with progressive pallor and diaphoresis. In all cases, post-mortem histopathological examination confirmed the diagnosis of amniotic fluid embolism, supported by special histochemical stains, specifically Alcian Blue (pH 2.5) and Dane stain [77, 78]. No additional immunohistochemical investigations were considered necessary.

#### Illustrative case

A 35-year-old primiparous woman (81 kg, 167 cm), at term, was admitted following spontaneous rupture of membranes. She suddenly developed pallor, with immediate loss of consciousness and bradycardia, and died shortly thereafter.

**Fig. 7:**
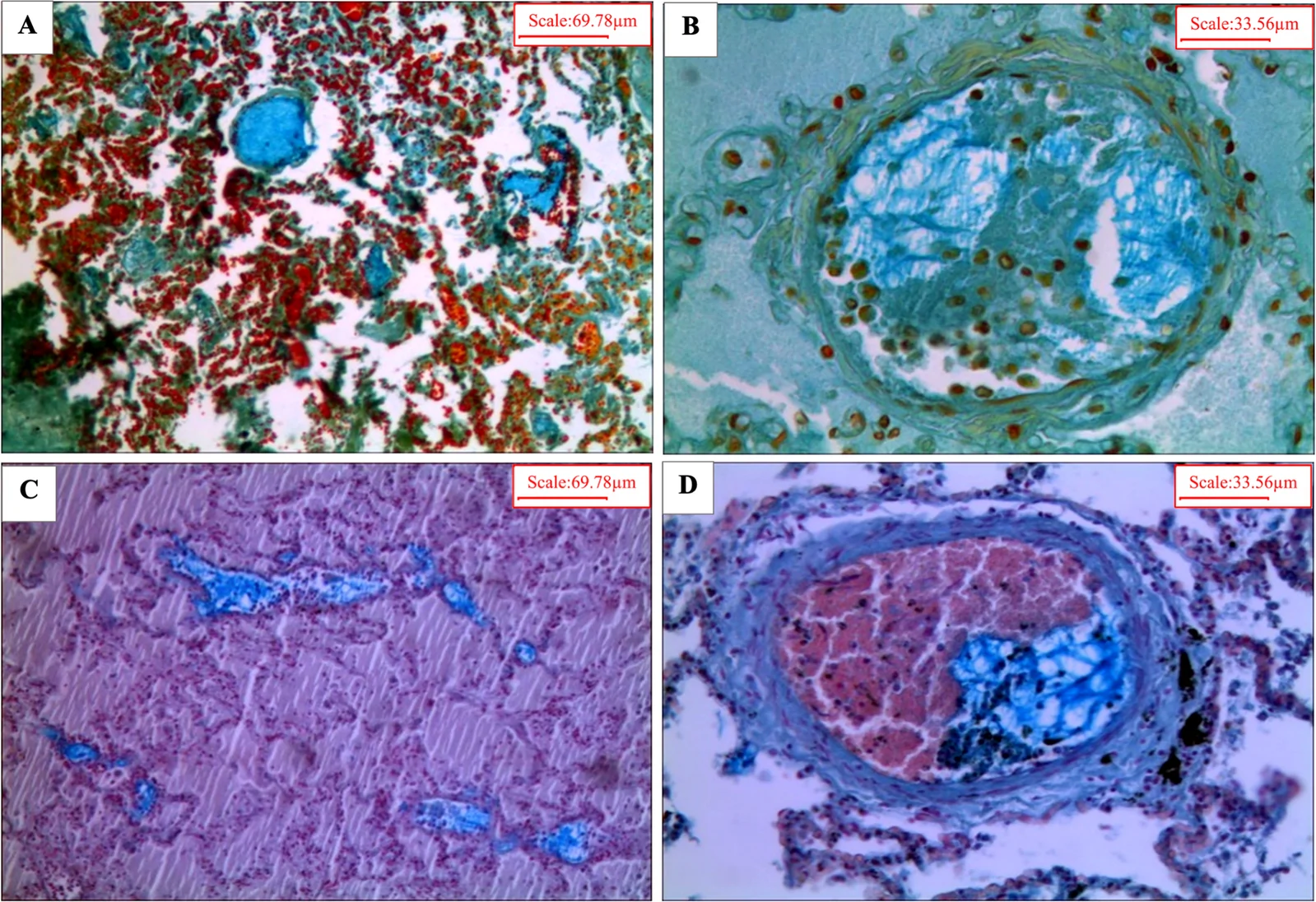
Microscopic images of lung sections showing amniotic fluid embolism involving pulmonary vessels, capillaries, and alveolar septa. In A-B (Dane–Herman stain, 100× and 200×): keratin and prekeratin appear red–orange, mucin turquoise, and nuclei dark brown–orange. In C-D: (Alcian Blue pH 2.5, 100× and 200×): positive staining for mucin (turquoise)

At autopsy, the lungs appeared enlarged and increased in weight (right lung 490 g; left lung 410 g), markedly congested and edematous, with numerous diffuse subpleural petechiae. Histopathological examination revealed diffuse amniotic fluid embolism, with embolic material composed of poorly cellular mucoid substance within small-caliber pulmonary vessels and capillaries of the alveolar septa, extending to subpleural regions. The embolic material stained positively with Dane–Herman stain, demonstrating mucus, keratin, and prekeratin, with anucleated squamous cells stained red-orange (Fig. 7A and B), and with Alcian blue stain (pH 2.5), confirming the presence of mucin (Fig. 7C and D).

At the conclusion of all investigations, the cause of death was identified as massive amniotic fluid embolism.

### Pulmonary tumor embolism (PTE)

Pulmonary tumor embolism (PTE) consists of the occlusion of pulmonary vessels by tumor cells [107] and may occur through different pathogenetic mechanisms [41]: (i) embolization of a distant primary tumor to the pulmonary arteries without direct vascular invasion (typically presenting as macroembolism); (ii) intravascular growth of malignant cells within pulmonary arteries (microembolism); (iii) direct invasion and obstruction of pulmonary vessels by a primary or secondary lung tumor [4]; (iv) a combination of these mechanisms [108]. Tumor dissemination occurs mainly via the hematogenous route, through which neoplastic cells reach the pulmonary circulation, leading to vascular occlusion, metastatic spread, lymphatic invasion, and the development of pulmonary hypertension. These processes are mediated by complex and not yet fully clarified interactions [109] involving molecular pathways regulating angiogenesis, apoptosis, and inflammation [1, 110, 111]. The pathogenesis of PTE has been explained by two main mechanisms: (i) vascular remodeling secondary to endothelial dysfunction induced by tumor cells, and (ii) increased pulmonary vascular resistance due to mechanical obstruction of the arterial bed by tumor emboli, resulting in impaired adaptive capacity of the pulmonary circulation [112].

Autopsy studies report an incidence ranging from 3% to 26% among patients with solid tumors [108, 109, 113]. In most cases, emboli originate from extrapulmonary malignancies [2], including breast, gastric [112], renal, hepatic, and urothelial carcinomas, as well as choriocarcinoma and sarcoma [114]. Pancreatic cancer, particularly in the early phases and in the presence of metastases [110, 115], is frequently associated with migratory thrombophlebitis (Trousseau’s syndrome) [115], which may predispose to pulmonary thromboembolism [116] or deep vein thrombosis.

Clinically, PTE typically presents with progressive dyspnea, cough, hypoxia, and pulmonary hypertension – often severe – potentially leading to subacute cor pulmonale [116]. Diagnosis is challenging, as imaging findings are frequently non-specific. Contrast-enhanced computed tomography may reveal persistent filling defects in the pulmonary arteries that do not resolve with anticoagulant or fibrinolytic therapy [41]. However, these findings may mimic conventional thromboembolic disease or, in cases of microscopic embolization, may even be absent, making ante-mortem diagnosis particularly difficult [2]. Treatment is primarily directed at the underlying malignancy, often with palliative intent, and the prognosis remains poor, with survival typically limited to a few weeks from symptom onset [116]. In some cases, PTE may represent the first manifestation of an occult malignancy [117, 118]. Although a definitive causal relationship between cancer and thromboembolic events has not been fully established, such events may precede the clinical diagnosis of malignancy and act as early indicators of an underlying neoplasm [119].

Antemortem diagnosis is difficult and is therefore most often established post-mortem [108, 120]. In forensic practice, PTE represents a potential cause of sudden unexpected death, particularly in patients with undiagnosed malignancy [121]. The identification of tumor emboli showing adhesion to the vascular wall, often associated with fibrin deposition, supports ante-mortem hematogenous dissemination requiring active circulation and contributes to establishing the mechanism of death.

#### Results of the retrospective evaluation and illustrative case

The retrospective analysis identified only one case, reported below.

*An 80-year-old man (83 kg*,* 155 cm)*,* with a history of moderately differentiated pulmonary adenocarcinoma under clinical follow-up*,* suddenly became ill while walking outdoors and collapsed. He was promptly assisted and transported to hospital but died a few hours later during ongoing diagnostic evaluation.*

**Fig. 8:**
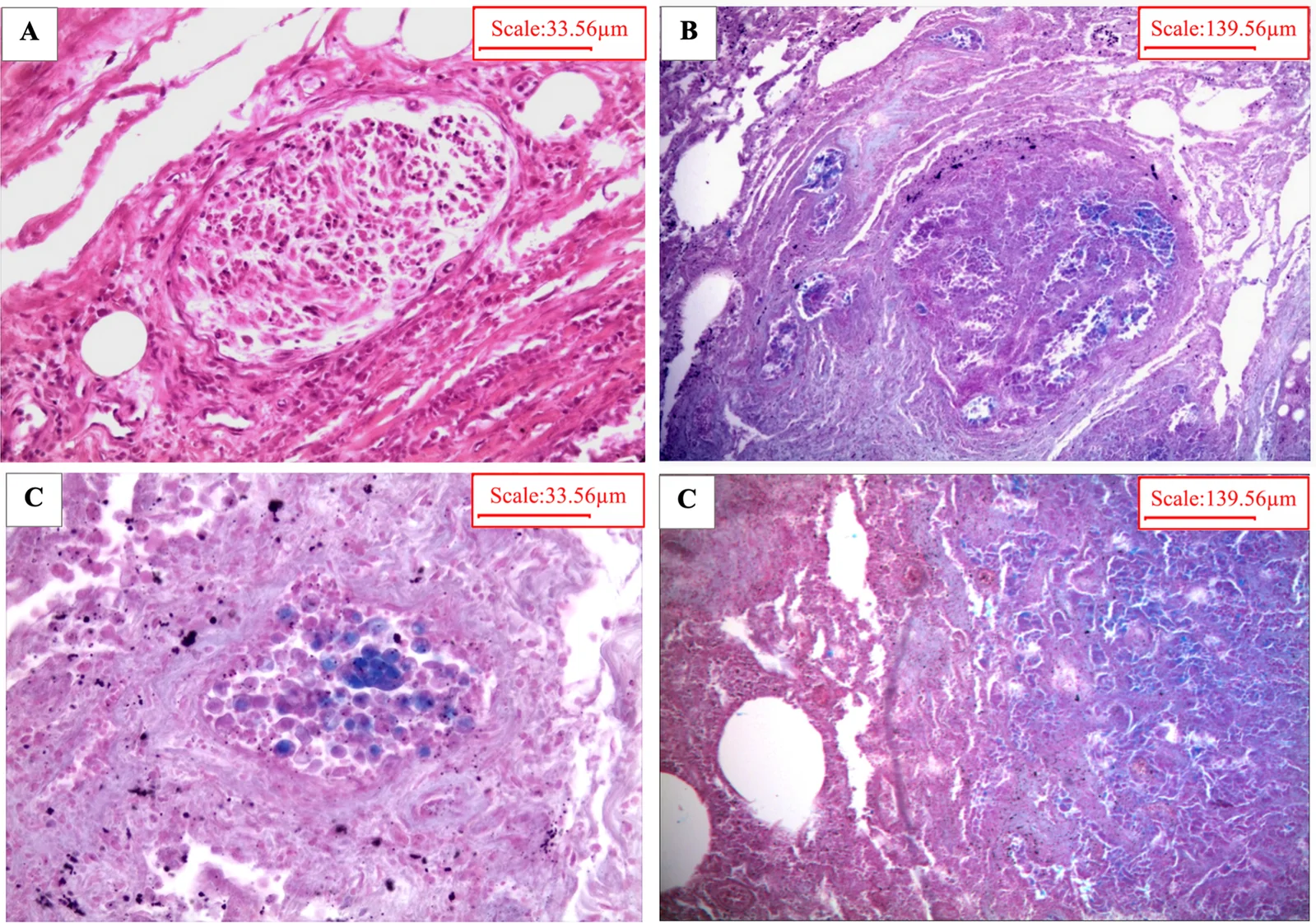
Microscopic images of lung sections showing neoplastic pulmonary embolism and the corresponding primary pulmonary adenocarcinoma. In A (H&E, 200×): neoplastic embolus within a pulmonary vessel, composed of moderately differentiated adenocarcinoma cells. In B–C (Alcian Blue pH 2.5, 50× and 200×): abundant mucin production by the neoplastic emboli. In D (Alcian Blue pH 2.5, 50×): primary pulmonary moderately differentiated adenocarcinoma showing a nodular growth pattern with morphological features corresponding to the neoplastic emboli

At autopsy, the lungs appeared enlarged, with nearly normal weight (right lung 530 g; left lung 470 g), congested and edematous, with scattered subpleural petechiae. The right lung parenchyma showed a nodular lesion of increased consistency, with a lardaceous appearance and poorly defined margins. Histopathological examination revealed numerous foci of neoplastic embolism within pulmonary vessels, composed of moderately differentiated adenocarcinoma cells (Fig. 8A). The neoplastic emboli showed abundant mucin production, confirmed by Alcian blue staining at pH 2.5 (Fig. 8B and C). Histological examination also demonstrated the known primary pulmonary adenocarcinoma within the lung parenchyma, showing a moderately differentiated nodular growth pattern (Fig. 8D), with metastatic involvement of the peribronchial lymph nodes. The tumor infiltrated the wall of a medium-caliber bronchus, including the mucosa, with focal protrusion into the bronchial lumen. The close morphological correspondence between the primary tumor and the neoplastic emboli supported the diagnosis of pulmonary neoplastic embolism.

At the conclusion of all investigations, the cause of death was identified as massive neoplastic pulmonary embolism.

### Air embolism (AE)

Air embolism (AE) is a relatively rare and often underrecognized condition [122], caused by the entry of gas into the vascular system. This may occur through the introduction of air or medically used gases – such as carbon dioxide, nitrous oxide, or nitrogen [123] – or as a result of rapid changes in ambient pressure, leading to the formation of gas bubbles from dissolved blood gases [124]. Two conditions are required for the development of AE: a direct communication between the external environment and the vascular system, and a sufficient pressure gradient to allow gas entry. AE may therefore occur in a variety of settings, including diving accidents and numerous iatrogenic medical procedures [125]. From a medico-legal perspective, venous air embolism has also been reported following penetrating injuries, gunshot wounds to the head, severe craniofacial trauma involving the dural venous sinuses, and incised wounds of the neck. In these situations, a direct communication between the atmosphere and non-collapsible venous structures, such as the dural venous sinuses or large cervical veins, allows the entrainment of air into the venous circulation under the effect of negative intrathoracic pressure during spontaneous inspiration, potentially resulting in fatal air embolism [126, 127]. Reported procedures include percutaneous lung biopsy or tumor ablation [128, 129], arterial catheterization, cardiopulmonary bypass, pressurized intravenous infusions [130], catheter manipulation [131], contrast injection [132], laparoscopy [133], endoscopy [134], and endoscopic retrograde cholangiopancreatography (ERCP) [135]. In the specific context of ERCP, risk factors include previous liver surgery, hepatic trauma, intrahepatic abscess, biliary tract surgery, the presence of prostheses or stents, cholangitis [136, 137], liver tumors [138], and anatomical abnormalities such as hepatobiliary fistulas [139] or intrahepatic and extrahepatic leaks [140].

Based on the site of gas entry and embolization, AE is classified into venous and arterial forms [125]. Venous AE occurs when gas enters the systemic venous circulation [141] and is transported to the pulmonary arteries, potentially resulting in acute right heart failure [142]. Arterial AE, even when caused by small volumes of gas, involves the systemic circulation and may lead to occlusion of terminal arteries with ischemia of vital organs, including the brain, spinal cord, heart, kidneys, spleen, and gastrointestinal tract. Cerebral and coronary arterial gas embolism are particularly lethal due to the high sensitivity of these organs to hypoxia [143, 144]. In the presence of abnormal vascular communications - such as a patent foramen ovale, intrapulmonary shunts, or retrograde venous flow through the superior vena cava or vertebral venous system [135] - gas emboli may bypass pulmonary filtration and enter the arterial circulation, resulting in paradoxical embolism. While small venous emboli are often clinically tolerated, even minimal paradoxical emboli may have fatal consequences [145]. Clinical manifestations vary depending on the affected vascular territory and may include chest pain, dyspnea, cyanosis, hypotension, and neurological deficits such as confusion or reduced consciousness, potentially progressing to cardiac arrest and death [125]. Immediate management consists of high-flow oxygen administration and appropriate patient positioning (Trendelenburg or lateral decubitus), while definitive treatment is represented by hyperbaric oxygen therapy, which improves tissue oxygenation and reduces ischemia–reperfusion injury [122, 146].

Antemortem diagnosis relies primarily on imaging techniques, particularly computed tomography (CT), which allows direct visualization of intravascular gas and remains the imaging modality of choice in suspected air embolism. In the post-mortem setting, diagnosis may be challenging, especially in the absence of post-mortem CT performed within 48 h after death [147], owing to progressive gas reabsorption and the difficulty in distinguishing true intravascular gas from putrefactive changes. In such cases, invasive techniques such as cardiac puncture or underwater dissection may be required, although accurate quantification of intravascular gas remains difficult [148].

Although rare, cases of self-induced air embolism for suicidal purposes have been reported, including intravenous injection of air combined with sedative agents such as midazolam [149].

From a medico-legal perspective, the diagnosis of fatal air embolism requires not only the demonstration of intravascular gas but also evidence that gas entry occurred during life. When ante-mortem radiological documentation is unavailable, gas chromatographic analysis of intracardiac gas [150, 151] and the histological demonstration of platelet and leukocyte aggregates surrounding intravascular gas spaces [152] – although rarely addressed in the current literature – may represent useful forensic tools for supporting the diagnosis of ante-mortem air embolism.

#### Results of the retrospective evaluation and illustrative case

The retrospective analysis identified a single case, reported below.

*A 51-year-old woman (75 kg*,* 158 cm)*,* with a history of recurrent biliary and pancreatic colic*,* had previously undergone cholecystectomy*,* extracorporeal shock wave lithotripsy*,* and endoscopic retrograde cholangiopancreatography (ERCP) with sphincterotomy for intrahepatic biliary stones. Due to persistence of a large stone*,* a second ERCP was scheduled. During the procedure*,* she developed acute respiratory and cardiac complications and died two hours later. Emergency computed tomography revealed massive intracardiac air and cerebral gas embolism.*

**Fig. 9:**
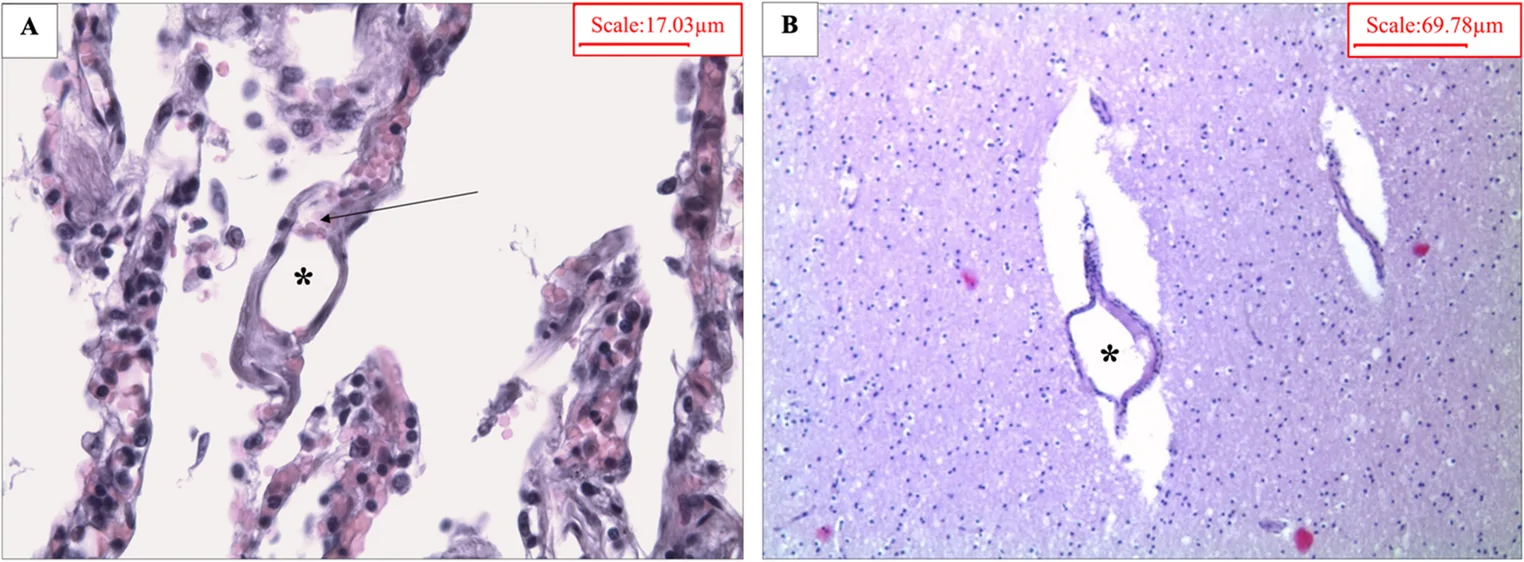
Microscopic images of lung and brain sections showing air embolism. In A (H&E, 400×): alveolar capillaries appearing partially empty (asterisk), with peripheral displacement of erythrocytes adherent to the vessel wall (arrow). In B (H&E, 100×): Brain section: arterial vessel appearing optically empty (asterisk), with perivascular fixation artifacts

At autopsy, a small gas bubble was identified within a leptomeningeal vein in the left occipital region. A biliary–vascular fistula, not appreciable on direct inspection of the biliary tree, was demonstrated by air leakage from the suprahepatic veins following insufflation of the common bile duct. A patent foramen ovale (PFO) was also identified. The lungs appeared enlarged, with approximately normal weight (right lung 410 g; left lung 355 g), congested and edematous, with few subpleural petechiae. Histopathological examination revealed dilated alveolar capillaries appearing either optically empty, without evidence of thrombi or other intravascular material, or containing erythrocytes displaced toward the periphery and adherent to the vessel wall, in association with pulmonary edema (Fig. 9A). In the brain, small optically empty intravascular microvesicles were observed (Fig. 9B), occasionally displacing residual blood elements toward the vessel periphery. Although histology cannot directly demonstrate intravascular gas, these findings were interpreted in conjunction with the ante-mortem CT findings and the autopsy demonstration of a biliary–vascular fistula as supportive of the diagnosis of gas embolism. At the conclusion of all investigations, the cause of death was identified as pulmonary and cerebral gas embolism based on the integration of the ante-mortem CT findings, the autopsy demonstration of a biliary–vascular fistula and a gas bubble within a leptomeningeal vein, together with the supportive histopathological findings. The biliary–vascular fistula provided the anatomical route for venous air entry during ERCP, whereas the patent foramen ovale most likely accounted for the paradoxical cerebral embolization.

## Other forms of non-thrombotic pulmonary embolism

A review of the literature identified additional forms of non-thrombotic pulmonary embolism (NTPE) that were not observed in our 30-year autopsy series. Although absent in the present cohort, these entities warrant consideration due to their potential forensic relevance. For the sake of completeness, a concise overview of these less frequently encountered forms of NTPE is provided below.

### Hydatid embolism

Hydatid disease (echinococcosis) is a parasitic zoonosis acquired through ingestion of *Echinococcus granulosus* eggs in contaminated food or water. After ingestion, the larvae penetrate the gastrointestinal mucosa and give rise to hydatid cysts [153]. These are primarily distributed via the portal circulation to the liver and, subsequently, to the lungs through the pulmonary artery, although other sites such as the spleen, muscles, and central nervous system may also be involved. Rupture of hydatid cysts [154] may result in the release of cystic and vesicular material into the vascular system [155, 156], leading to hematogenous dissemination. Intravascular rupture can cause mechanical obstruction of pulmonary or systemic vessels, chronic pulmonary hypertension, and, in severe cases, sudden death [157].

### Filler embolism

Hyaluronic acid (HA) is a glycosaminoglycan widely distributed in connective, epithelial, and nervous tissues, representing a key component of the extracellular matrix [158]. Due to its viscoelastic, hydrophilic, and non-immunogenic properties, it is extensively used in aesthetic and reconstructive procedures, as well as in pharmaceutical applications. Although generally considered safe [159], embolic complications associated with HA injections have been increasingly reported. These events most commonly involve the ophthalmic artery and its branches (*n* = 18), but have also been described in small subcutaneous vessels (*n* = 9), the central retinal artery with secondary cerebral infarction (*n* = 2), and more rarely in the deep lingual, superficial temporal, and facial arteries (*n* = 1 each). Additional cases have been reported following genital cosmetic procedures, including vaginoplasty or vaginal tightening [160], the latter also performed using polyacrylamide gel [161], as well as male procedures for penile or scrotal augmentation [162]. Despite being considered minimally invasive, these procedures may result in severe and potentially fatal complications, including pulmonary embolism [159]. In one study [160], approximately 60% of patients developed respiratory symptoms such as dyspnea and chest pain within 12 h of HA injection, with several cases progressing to multiorgan failure and death. Histopathological examination demonstrated diffuse embolization of an amorphous basophilic material within small pulmonary vessels, which stained positively with Alcian blue, allowing attribution of death to HA-related pulmonary embolism [160].

Silicone represents another material associated with embolic phenomena. Although historically considered immunologically inert, it has been shown to induce inflammatory responses, with IgG binding to silicone particles [163]. Histologically, this is characterized by inflammatory infiltrates and granuloma formation with vacuolated spaces containing silicone deposits. While silicone implants are approved for clinical use [164], injectable liquid silicone – used illicitly for soft tissue augmentation (e.g., hips, buttocks, breasts, face, and genital regions) – is associated with a high risk of migration and embolization, sometimes resulting in fatal outcomes [165, 166].

### Gastrointestinal embolism

Gastrointestinal embolism represents an extremely rare group of pathological entities characterized by heterogeneous but related etiopathogenetic mechanisms. Clinical and radiological findings are often atypical [167], contributing to diagnostic difficulty and high mortality, with autopsy frequently playing a decisive role. Most cases are associated with abnormal communications between the gastrointestinal tract and the vascular system. These may be direct, such as between the esophagogastric region and the heart [168, 169], or involve enterovenous fistulas, including duodenal ulcers fistulizing into the inferior vena cava [170, 171]. In such settings, histological examination may reveal PAS-positive material, vegetable fibers, and biliary components within pulmonary and systemic vessels, likely related to altered pressure gradients [167]. In traumatic contexts, gastrointestinal embolism has been described even in the absence of overt macroscopic fistulas, with pulmonary vessels containing alimentary material such as vegetable fibers and PAS-amylase-positive polysaccharides, sometimes associated with muscle fragments [172]. This phenomenon is thought to result from critical increases in intraluminal pressure facilitating the entry of enteric contents into the circulation. Pulmonary embolism due to food material is exceptionally rare, with only eight cases reported since its first description in 1961 [167, 173], and may also present as systemic embolization involving organs such as the heart, liver, spleen, and kidneys [174].

Fecal embolism, first described in 1978 [175], has been reported in association with ileal diverticulosis and portosystemic circulation, where emboli of calcified fecal material were identified within pulmonary arterioles, likely originating from ileovenous fistulas.

A related entity involves the biliary system. In the presence of hepatobiliary disease or trauma, pathological communications between the biliary tract and the venous system may allow bile to enter the circulation, resulting in bile pulmonary embolism (BPE) [176]. First described in 1952 [177], BPE is an exceptionally rare condition that may be incidental or clinically severe [178]. Histologically, it is characterized by occlusion of small pulmonary vessels by bile material, often associated with hemorrhagic infarction.

### Embolism following ureteral lithotripsy

The literature reports only two cases of fatal embolism following ureteral stone fragmentation by lithotripsy: one associated with laser lithotripsy [179] and the other with percutaneous pneumatic lithotripsy [180]. Although these minimally invasive techniques are generally considered safe and represent the gold standard for the treatment of complex ureteral stones [179], they are not devoid of complications. Reported adverse events include hemorrhage (the most common), perforation, mucosal injury, infection, damage to adjacent organs, and migration of stone fragments [181, 182].

In the case related to laser lithotripsy, performed in a patient with ureteral stents and urinary catheterization, a rare complication occurred, consisting of microembolization of calcified material into both pulmonary and systemic circulation, with deposition of stone fragments in medium- and large-caliber pulmonary arteries and in the myocardium. This event led to disseminated intravascular coagulation (DIC) approximately two hours after the procedure [179].

In the second case, involving percutaneous pneumatic lithotripsy in a patient with bilateral hydronephrosis and multiple calyceal stones, sudden death occurred approximately three hours after the procedure due to cardiac arrest. Although no macroscopic embolism was identified at autopsy, histological examination revealed numerous microcalculi within pulmonary arterioles and capillaries, composed of calcium salts and confirmed by von Kossa staining [180]. The exact pathogenetic mechanism remains unclear. It has been hypothesized that stone fragments may enter damaged vessels during the procedure, embolize the pulmonary circulation, and induce increased pulmonary vascular resistance, hypoxemia, and reduced cardiac output, ultimately leading to cardiac arrest and sudden death.

### Trophoblastic and decidual embolism

Trophoblastic embolism (TE) is a rare and potentially fatal complication of gestational trophoblastic disease (GTD), characterized by the entry of abnormal placental trophoblastic cells into the maternal circulation. These include type I cells (intact trophoblasts) and type II cells (amorphous trophoblastic fragments) [183], typically originating from the site of placental implantation [1]. TE may occur subclinically during normal pregnancy, become more pronounced in pathological conditions such as eclampsia, and reach a massive extent in hydatidiform mole [1]. Despite this, fatal outcomes are rare [184, 185]. TE may also be associated with decidual embolism, in which maternal decidual tissue enters the venous circulation from the placental implantation site or decidualized endometrium, particularly in the context of pregnancy, delivery, abortion, or obstetric procedures. Only a limited number of fatal cases have been reported in the literature, including a 15-year-old patient at 11 weeks of gestation [186] and a 42-year-old woman following induced abortion [187]. In both cases, autopsy demonstrated massive pulmonary embolization, with additional cerebral involvement in the latter. Histopathological findings were characterized by extensive syncytiotrophoblastic embolism of the pulmonary microcirculation, associated with a marked placental reaction.

### Embolism due to inorganic material and foreign bodies

Inorganic particulate matter and foreign bodies represent rare causes of non-thrombotic pulmonary embolism (NTPE), but may lead to severe clinical consequences [3].

Among the most common iatrogenic sources are peripheral and central venous catheters, widely used in clinical practice. Catheter fracture or transection – particularly involving the tip – may result in embolization of fragments, with an estimated incidence ranging from 0.2% to 4.2% in adults and 1.4% to 3.6% in children [3, 188]. In addition, fibrin sheath formation around the catheter may occur; when calcified, this can lead to mural or intraluminal thrombi and, more rarely, to NTPE due to fragmentation of the sheath [189].

Radiotherapy seeds, small radioactive implants composed of iodine-125 or palladium-103 used in the treatment of prostate and urethral cancers, may also embolize to the pulmonary circulation [190, 191], although the underlying mechanism remains unclear. In such cases, removal is generally discouraged, as the risks associated with embolectomy exceed those of leaving the material in situ [192].

Polymethylmethacrylate (PMMA), used in percutaneous vertebroplasty for vertebral fractures, may extravasate during injection – an event reported in up to 24% of cases – and migrate into the venous system, occasionally reaching the pulmonary circulation and causing potentially fatal embolism [193]. In some cases, PMMA embolism may coexist with fat embolism, likely due to displacement of bone marrow and adipose tissue during the procedure [194].

Finally, intravenous drug use represents a further source of foreign material embolism [1]. Substances are often crushed and injected in suspension, allowing particulate matter such as talc, starch, cellulose, and cotton fibers to reach the pulmonary microcirculation, where they may induce foreign body granulomatous reactions in the perivascular interstitium [195, 196].

## Discussion

Non-thrombotic pulmonary embolisms (NTPE) represent a heterogeneous and still underrecognized group of embolic conditions, characterized by complex pathogenetic mechanisms, variable clinical presentations, and significant diagnostic challenges in both clinical and post-mortem settings. The findings of this 30-year forensic autopsy study confirm that NTPE, although relatively uncommon (9.8% of all fatal pulmonary embolisms), constitute a relevant cause of sudden and unexpected death, often occurring in the absence of specific or pathognomonic macroscopic findings. The distribution of cases observed in our cohort highlights the predominance of trauma-related and iatrogenic conditions, with bone marrow embolism representing the most frequent subtype, followed by septic and fat embolism. This pattern underscores the central role of external triggers and medical or surgical interventions in the pathogenesis of NTPE, particularly within a forensic context. Notably, several cases were associated with routine or elective procedures, emphasizing the potential for unexpected fatal outcomes even in apparently controlled clinical settings.

A key finding of this study is the limited diagnostic value of macroscopic examination alone. In the majority of cases, definitive diagnosis relied on histopathological evaluation, often supported by specific histochemical staining techniques. This confirms that NTPE are likely underdiagnosed or misinterpreted when autopsy investigations are incomplete or when microscopic analysis is not systematically performed. The identification of embolic material - whether cellular, infectious, lipidic, neoplastic, or gaseous - requires targeted investigation and a high level of expertise, highlighting the central role of forensic pathology in these cases.

In this context, the forensic autopsy represents the diagnostic gold standard for NTPE. However, the progressive decline in autopsy rates reported in the literature [197, 198] raises concerns about a potential underrecognition of these entities and a consequent loss of critical epidemiological and diagnostic information [199]. This aspect is particularly relevant for NTPE, in which diagnosis frequently depends on histopathological confirmation and may not be achievable through clinical or radiological assessment alone. Therefore, a comprehensive and multidisciplinary approach – including macroscopic examination, histopathology, histochemistry, and, when indicated, radiological and toxicological analyses – is essential to ensure accurate identification and interpretation.

From a medico-legal perspective, NTPE assume particular relevance due to their frequent association with medical procedures, traumatic events, and high-risk clinical conditions such as pregnancy, malignancy, or severe infection. In this setting, accurate post-mortem diagnosis is crucial not only for determining the cause of death but also for reconstructing the sequence of events and evaluating the appropriateness of medical management. The occurrence of NTPE in relation to invasive or routine procedures – including orthopedic surgery, ERCP, cosmetic interventions, or resuscitative maneuvers – further emphasizes the importance of careful forensic assessment, particularly in cases where medical liability may be considered.

This study presents several strengths, including the long observation period, the relatively large autopsy-based cohort, and the integration of histopathological, clinical, and circumstantial data, allowing a comprehensive characterization of NTPE within a real-world forensic setting. Although conducted at a single institution, the present investigation ensured methodological consistency across all cases throughout the entire 30-year study period, including standardized autopsy procedures, histopathological sampling protocols, and diagnostic criteria, thereby minimizing inter-center variability. Nevertheless, some limitations should be acknowledged. The retrospective design may have introduced selection bias and limited the availability of complete clinical and circumstantial information in some cases. In addition, the single-center setting may reduce the generalizability of the findings to different forensic or healthcare systems, while the relatively small number of cases for certain NTPE subtypes reflects the intrinsic rarity of these conditions and limits the possibility of more advanced statistical analyses. Future multicenter studies, based on standardized forensic and histopathological protocols, would be valuable to better define the epidemiology, risk factors, and pathogenetic mechanisms of NTPE, as well as to improve their clinical and post-mortem recognition.

In conclusion, non-thrombotic pulmonary embolisms represent a rare but clinically and forensically significant cause of death, characterized by heterogeneous etiologies, non-specific presentation, and marked diagnostic complexity. They frequently arise in traumatic, iatrogenic, and procedural contexts and may lead to sudden and unexpected death, often in the absence of specific macroscopic findings. Histopathological examination is therefore essential for diagnosis, as embolic material is frequently undetectable at gross examination. A systematic and multidisciplinary forensic autopsy approach remains crucial to accurately define the cause and mechanisms of death. From a medico-legal perspective, accurate recognition of NTPE is particularly relevant in procedure-related cases, where it contributes to the evaluation of clinical management and potential healthcare responsibility. Strengthening the role of autopsy and enhancing awareness of NTPE among clinicians and forensic practitioners may significantly improve diagnostic accuracy and contribute to a more reliable interpretation of sudden and unexpected deaths.

## Data Availability

No datasets were generated or analysed during the current study.
